# TEMPO-Oxidized Spruce Galactoglucomannan–Biopolymer with Enhanced Antioxidant Activity and Selective Heavy-Metal Sorption

**DOI:** 10.3390/antiox14050569

**Published:** 2025-05-09

**Authors:** Vladislav A. Ionin, Yuriy N. Malyar, Valentina S. Borovkova, Dmitriy V. Zimonin, Aleksandr S. Kazachenko

**Affiliations:** 1 Institute of Chemistry and Chemical Technology, Krasnoyarsk Science Center, Siberian Branch Russian Academy of Sciences, Akademgorodok 50/24, Krasnoyarsk 660036, Russia; ionin.va@icct.krasn.ru (V.A.I.); bing0015@mail.ru (V.S.B.); zimonind89@mail.ru (D.V.Z.); askazachenko@sfu-kras.ru (A.S.K.); 2 School of Non-Ferrous Metals, Siberian Federal University, pr. Svobodny 79, Krasnoyarsk 660041, Russia; 3 Institute of Chemical Technologies, Reshetnev Siberian State University of Science and Technology, Mira St 82, Krasnoyarsk 660049, Russia

**Keywords:** galactoglucomannan, oxidation, 2,2,6,6-tetramethylpiperidin-1-oxyl, heavy-metal adsorption, 1,1-diphenyl-2-picrylhydrazyl, hydroxyl radicals

## Abstract

This study examines galactoglucomannan, a well-studied biopolymer isolated from Siberian spruce (*Picea obovata* Ledeb). Due to its structure, abundant with hydroxyl groups, galactoglucomannan’s properties, such as heavy-metal ion affinity, are considered to be mediocre. Nevertheless, there are various ways to enhance its functionality via oxidative TEMPO/NaBr/NaOCl processing. This work is concerned with the determination of the oxidation effect on the structure and performance properties, such as thermal decomposition behavior, antioxidant activity, and selective heavy-metal sorption. In the results, TEMPO-oxidized galactoglucomannan yields vary in the range of 78.3 ± 6.4 wt.%. The carboxylate group in the oxidized derivative represents up to 0.084 g/1 g of the sample. According to antioxidant activity tests, the oxidized galactoglucomannan exceeds the initial sample in terms of hydroxyl radical scavenging ability. The spectral characteristics of the initial and oxidized galactoglucomannan samples reveal the differences in absorption units (1725, 1610, and 1371 cm^−1^). The preservation of the polymeric structure was confirmed by the gel permeation chromatography analysis results. The heavy-metal ion capacity of galactoglucomannan is higher for the oxidized derivative, which demonstrated Cd^2+^, Fe^2+^, Cu^2+^, and Pb^2+^ adsorption values of 166.8 mg/g, 142.8 mg/g, 150.0 mg/g, and 199.2 mg/g, accordingly. The obtained result of the competitive heavy-metal ion adsorption of oxidized galactoglucomannan also exceeds its initial form, as characterized by its summary 143.1 mg/g capacity.

## 1. Introduction

In nature, heavy metals (HMs) such as cadmium, lead, copper, or iron occur at low concentrations depending on the source of the matter, or due to the consequences of weathering, volcanic activities, etc. [1,2,3]. Nevertheless, with the increasing role and scale of anthropogenic activities around the world, which produce approximately 70% of global waste [3], a redistribution of resources takes place, leading to local increases in pollutant concentrations, which result in the contamination of soils used by humanity to raise crops [4]. Even low concentrations of heavy metals easily absorbed by plant cells make an impact on plant growth and development [5]. Therefore, plants, in the course of evolution, developed a defensive mechanism, aiding to avoid the negative effects of heavy metals, by the immobilization or accumulation of heavy metals in cells by forming chelating complexes or by binding with polysaccharides and other biologically active compounds [6].

The recent achievements in the chemical modification of polysaccharides are dedicated to improving functional properties via the mild, non-aggressive intrinsic tuning structure of macromolecules with a varied degree of complexity [7]. Previous successes in the synthesis of sulfated polysaccharide derivatives with a 12–13% sulfur content could have been promising due to their pronounced high antioxidant abilities [8,9]. The preservation of inherited anticoagulant, antimicrobial, and antitumor effects on the physiological functions influenced by the initial polysaccharides’ biological properties has been described earlier [10,11,12,13]. Despite sulfated polysaccharides having some favorable properties due to their biocompatibility, antioxidant activity, and reduced toxicity [14], the sulfation commonly employs harsh conditions for the reaction to take place, affecting the polysaccharides’ degradation due to the decay of vulnerable linkages [15,16]. Upon such modifications, the sulfated polysaccharides’ physical and biological properties are difficult to manage because they undergo alterations [17].

On the other hand, one of the most promising directions for modification is the regioselective oxidation of the primary hydroxyls of polysaccharides using 2,2,6,6-tetramethylpiperidin-1-oxyl (TEMPO) [18,19,20], which could enhance the suitability of the properties of polysaccharides for various applications, such as water solubility and emulsification abilities in synthesis [21,22]. In addition to the abovementioned aspects, as a result of TEMPO-catalyzed oxidation the neutral basis of the original polysaccharide is altered to a highly negatively charged state, due to the conversion of the primary hydroxyls into carboxylates, impacting the affinity with HM ions, which could be subsequently used in the development of novel adsorptive functional materials, such as hydrogels and composite films [23,24].

Here, we focus on one of the common types of hemicelluloses obtained from softwoods—galactoglucomannan (GGM). This neutral polysaccharide’s structure is represented mainly by β-D-mannopyranoses (Man*p*), commonly substituted by α-D-glucopyranosic (Glc*p*) and β-D-galactopyranosic (Gal*p*) units, linked through β-(1→4) glycosidic linkages [25]. GGM’s solubility and chemical activity depend on different ratios of substitution by these units, affecting its accessibility and high reactivity.

Thus, this study focused on the oxidation of galactoglucomannan obtained from Siberian spruce (*Picea obovata* Ledeb*)* wood via a TEMPO-catalyzed medium. The impact of the oxidation on the GGM’s molecular weight and process dynamics was evaluated by the gel permeation chromatography (GPC) method. The dynamics of oxidation were also followed by spectrophotometric and titration methods of determining the formation of uronic acids. The structural changes in the obtained samples were registered by Fourier transform infrared spectroscopy (FTIR) and nuclear magnetic resonance (NMR) spectroscopy. Important biological properties such as antioxidant activity (AOA) were determined using a model of free and hydroxyl radical compounds. Finally, the oxidized GGM’s derivative adsorption capacity of heavy-metal ions (Fe^2+^, Cu^2+^, Cd^2+^, or Pb^2+^) was examined to propose further applications.

## 2. Materials and Methods

### 2.1. Materials

In course of this study, the galactoglucomannan was isolated from Siberian spuce (*Picea obovata* Ledeb*)* wood. TEMPO, 1,1-diphenyl-2-picrylhydrazyl (DPPH), and carbazole (analytical grade) were purchased from Sigma-Aldrich (St. Louis, MO, USA). The other chemical reagents (acetic acid, hydrogen peroxide, solvents, and metal salts) used in this study were purchased from Khimreaktivsnab (AO “Khimreaktivsnab”, Ufa, Russian Federation).

### 2.2. Isolation of Spruce Galactomannan and Its Monosaccharide Structure

In this study, as an initial material, isolated spruce galactoglucomannan was used for the following oxidation. Isolation, in a similar manner to that carried out in an earlier study [26], was performed as follows: 10 g of air-dried spruce wood sawdust was loaded into a three-necked glass flask equipped with a reflux condenser and an overhead stirrer. Then, a mixture containing acetic acid (30 wt.%), hydrogen peroxide (6 wt.%), and distilled water (liquid/solid ratio—15) was added, heated up to 100 °C and stirred at 300 rpm for 3 h. The obtained hot solution was separated from the wood residue via filtration using a Buchner funnel. After the cooling, the solution was concentrated on a rotary evaporator to a 1/5 volume. The galactoglucomannan was precipitated with a fourfold greater volume of ice-cold ethanol (96%) under slow stirring. After the precipitation, the obtained mixture was kept in ice-cold conditions for 12 h. The obtained precipitate further separated by filtration and were then dried in an “Iney-6” (Iney, Moscow, Russia) freeze-dryer. After the drying, the precipitate was mechanically ground in a mortar. The resulting medium yields of the GGM consist of up to 10 wt.% from the raw biomass material.

The monosaccharide composition of the initial GGM was determined by following a commonly used method [27,28], conducting the sample hydrolysis with the following modification to obtain trimethylsilyl derivatives. These derivatives were further analyzed by gas chromatography (GC). A VARIAN-450 GC-system equipped with a flame ionization detector and a VF-624ms (30 × 0.32 mm) capillary column were used. The analysis of the derivatives was performed in helium as a carrier gas, through an injector heated up to 250 °C, with an initial column temperature of 50 °C (5 min), rising up to 180 °C at a rate of 10 °C/min with a following retention at 180 °C for 37 min. The peaks on the chromatogram were identified based on an internal standard sample—the sorbitol, in accordance with the retention times of the monosaccharide’s tautomeric forms.

### 2.3. Galactoglucomannan TEMPO-Catalyzed Oxidation

A TEMPO-catalyzed oxidation of the isolated galactoglucomannan was conducted in a stirred three-necked flask using techniques described in [18,29], with some improvements. In a typical procedure, 0.8 g of NaBr and 0.04 g of TEMPO were added to 2 g of the air-dried GGM dissolved in 520 mL of distilled water under constant stirring (400 rpm). The reaction started simultaneously with the gradual addition of 80 mL of a freshly prepared 15 wt.% NaOCl solution, followed by a mixed pH adjustment to 9.5 via a 1 M HCl solution. The reaction mixture temperature was constantly cooled at 5 °C by an ice bath and pH was maintained at 9.5 via the gradual addition of a 0.1 M NaOH solution every 15 min. Consumption of the NaOH solution was permanently monitored as a function of time. Once the consumption remains unchanged for more than 3 periods of time in a row, 0.3 g of NaBH_4_ was added into the reaction mixture to reduce the carbonyl byproducts and stirred for another 30 min. After that, to quench the oxidation, the reaction medium pH was adjusted to 3 by a 1 M HCl solution and 60 mL of ethanol, with the addition of 0.06 g of NaCl. An obtained aqueous solution was evaporated approximately to a volume of 80 mL under vacuum conditions and further dialyzed using a 46 mm width cut-off dialysis bag, MF-5030-46 (MFPI, Seguin, TX, USA), with a 3.5 kg/mol pore size for 24 h to transfer the low-molecular-weight byproducts and salt residues into deionized water. Finally, the obtained solution was then poured into a Petri dish and dried at 60 °C in an oven to a constant weight.

The GGM’s oxidation was controlled by portions of the reaction medium analysis using spectrophotometric and titration methods. To evaluate the impact of oxidation, air-dried samples of the initial and oxidized GGM derivative (GGM-T) were studied via FTIR, NMR spectroscopy, and GPC. AOA assays were performed using compounds modeling free DPPH and hydroxyl radicals. The ability of the GGM samples to adsorb heavy-metal ions was determined using ICP-MS.

### 2.4. Galactoglucomannan Hydrolysis and Spectrophotometric Determination of the Formed Uronic Acids

The evaluation of the uronic acids formed by the strong acid hydrolysis of an oxidizing GGM’s reaction media was performed based on the modified carbazole method [30] using an Ecoview UV 6900 spectrophotometer (Shanghai Mapada Instruments Co., Ltd., Shanghai, China) with a created calibration curve similar to that in a previous study [18]. The analysis procedure was conducted as follows:

A 1 mL sample of the oxidizing GGM reaction medium was placed in a test tube, after which 6 mL of a borate-sulfuric acid agent (0.0125 M solution of sodium tetraborate in concentrated sulfuric acid) was slowly added under constantly ice-cold conditions. After that, the sample was vortexed, cooled, and 0.5 mL of a 0.015 wt.% solution of the carbazole-ethanol reagent was added, after which it was thoroughly mixed again and incubated at 80 °C for 5–10 min until a stable color change occurred. Then, the tube was cooled to room temperature, followed by the spectrophotometric measurement of the sample (λ = 530 nm), zeroed on distilled water as a reference solution.

### 2.5. Determination of the Initial and Oxidized Galactoglucomannan Carboxylic Content with a Potentiometric Titration

The determination of the initial and TEMPO-oxidized GGM carboxyl groups was performed based on a potentiometric titration method [31], as follows: Firstly, 5 mL of 0.1 M NaCl and 1 mL of 0.01 M HCl aqueous solutions were added to 0.3 g of the initial and oxidized GGM samples, dissolved in 100 mL of deionized water. The obtained mixtures were continuously stirred at 300 rpm for 6 h at room temperature until a well-dispersed solution was obtained. Furthermore, it was titrated with a 0.01 M NaOH solution at a 0.1 mL/min rate to adjust the pH to 10, performed with a Radelkis pH-stat (Radelkis, Budapest, Hungary). To evaluate the carboxylate content in the studied samples, the following Dalton’s law equation was used:

C_1_V_1_ = C_2_V_2_, (1)

where V_1_ and V_2_—volumes of the titrated substance and titrant, respectively, mL; C_1_—concentration of the carboxylate groups, mM/L; and C_2_—the alkali concentration, mM/L.

### 2.6. Fourier Transform Infrared Spectroscopy

The IR spectra were recorded by using a Shimadzu IRTracer-100 FTIR spectrometer (Shimadzu, Kyoto, Japan). The recorded spectrum of pure ZnSe crystal absorption is hereinafter referred to as a background spectrum. The initial and oxidized GGM samples were placed on a crystal, completely covering its surface, and the sample’s IR spectra were recorded in the range of 4000–630 cm^−1^ with a 4 cm^−1^ shooting resolution and 32 repeating scans. The resulting spectrum is the difference between the sample and a background spectra, which were processed further by using the OPUS 7.5 software package (Bruker BioSpin, Rheinstetten, Germany).

### 2.7. Nuclear Magnetic Resonance Measurements

The GGM sample’s chemical structure was estimated by recording the ^1^H and^13^C nuclear magnetic resonance spectra by using a Bruker Avance III 600 spectrometer (Bruker BioSpin, Rheinstetten, Germany). Before the analysis, both the initial and oxidized samples were air-dried, ground, and completely dissolved in the D_2_O, and placed into a 5 mm NMR tube at room temperature.

### 2.8. Gel Permeation Chromatography

To determine the initial and oxidized GGM samples’ molecular weight characteristics (average molecular weights and polydispersity index (PD)), a multi-detector GPC/SEC Agilent 1260 Infinity II system (Agilent Technologies, Santa Clara, CA, USA) was used. The system was equipped with Agilent PL Aquagel-OH Mixed-M and Agilent PL Aquagel-OH-30 columns, using a 0.1 M NaNO_3_ solution combined with 250 ppm NaN_3_ dissolved in deionized water as a mobile phase. Before the analysis, the samples were dissolved in a mobile phase (5 mg/mL) and poured through a 0.45 µm Agilent PES membrane filter (Millipore, Burlington, MA, USA). Then, 100 µL of the studied sample was analyzed in an eluent flow with a 1 mL/min rate.

The calibration was performed using an Agilent EasiVial PEG/PEO polyethylene glycol standard (Agilent, Santa Clara, CA, USA). All of the collected data were processed by Agilent GPC/SEC MDS software version 2.2.

### 2.9. Thermogravimetric Analysis

A thermogravimetric analysis (TGA) of the initial and oxidized GGM samples was performed using a NETZSCH STA 449 F1 Jupiter simultaneous thermal analysis instrument (NETZSCH STA 449 F1 Jupiter instrument, Netzsch, Selb, Germany) in an argon medium. The sample was placed in an Al_2_O_3_ cylindrical crucible with a perforated cover and analysis started with a heating rate of 10 °C/min in a 30–700 °C temperature range. The protective and blowout gas flow rates were 20 and 50 mL per minute, respectively. An empty corundum crucible with a cover was used as a reference. The calibration of the instrument was conducted according to the specifications, with the use of reference substances supplied by the instrument data. The sample weight for the analysis was determined on a Sartorius BP121S analytical lab-scale digital balance. The measurement data were processed using NETZSCH Proteus Thermal Analysis 5.1.0 software supplied by the instrument.

### 2.10. Antioxidant Activity

#### 2.10.1. DPPH Radical Scavenging Assay

The ability of the initial and oxidized GGM samples to scavenge DPPH radicals was determined according to the method described in previous studies [18,26], with some improvements. The absorbance of the samples was measured at 517 nm against a blank, using an Ecoview UV 6900 spectrophotometer (Shanghai Mapada Instruments Co., Ltd., Shanghai, China).

The DPPH radical scavenging ability of the initial and oxidized GGM samples was determined using the following equation:

(2) $$\mathrm{DPPH}\;\mathrm{Radical}\;\mathrm{Scavenging}\;\mathrm{Ability}\;(\%)=\left(1-\frac{A_{S}-A_{B}}{A_{C}}\right)*100\%,$$

where A_C_ represents the absorbance of the DPPH solution without a sample, A_S_ is the absorbance of the test sample mixed with the DPPH solution, and A_B_ is the absorbance of the sample without the DPPH solution.

#### 2.10.2. Hydroxyl Radical Scavenging Assay

The ability of the initial and oxidized GGM samples to scavenge the hydroxyl radicals was determined as previously reported [18,26]. The absorbance of the mixture was measured at 510 nm against the blank.

The capability to scavenge the hydroxyl radical was calculated as follows:

(3) $$\mathrm{Hydroxyl}\;\mathrm{Radical}\;\mathrm{Scavenging}\;\mathrm{Ability}\;(\%)=\left(1-\frac{A_{S}-A_{B}}{A_{C}}\right)*100\%$$

where A_C_ is the absorbance of the mixture without the sample, A_S_ is the absorbance of the test sample mixed with a reaction solution, and A_B_ is the absorbance of the sample without a salicylic acid solution.

### 2.11. Heavy-Metal Affinity Tests

In a typical procedure, 20 mg of the dried initial or oxidized GGM sample (25 mg) was added to 10 mL of the aqueous solutions containing heavy-metal ions (Fe^2+^, Cu^2+^, Cd^2+^, or Pb^2+^) alone with a 500 mg/L concentration. The adsorption process was carried out in an oscillator at room temperature and 300 rpm for 1 h. Then, 20 mL of ice-cold ethanol was added to the aqueous solution. This was followed by the mixing and removal of the solid precipitate via centrifugation and decantation. The obtained solution was poured through a 0.45 μm PTFE membrane. Finally, ethanol was evaporated, and the remaining heavy-metal ion concentration in the solution was determined by using an Agilent 7900 ICP-MS spectrometer (Agilent Technologies, Santa Clara, CA, USA) with a He collision cell to eliminate atomic interference. The solutions without an additional dilution were injected into argon plasma as a dry aerosol. The analysis was performed using a SemiQuant mode with atomic-scale calibration with a tuning solution. Sensitivity was up to 5 ng/L.

The adsorption degree (AD) of the initial and oxidized GGM samples was calculated as follows:

AD (%) = (C_in_ − C_f_)/C_in_ × 100, (4)

where C_in_ (mg/L) and C_f_ (mg/L) are the initial and final concentrations of the investigated heavy-metal ions.

The heavy-metal ion adsorption capacity (AC) of both the initial and oxidized GGM samples (mg/g) were calculated using the following equation:

AC= (C_in_ − C_f_)V/m, (5)

where C_in_ (mg/L) and C_f_ (mg/L) are the initial and final concentrations of the investigated heavy-metal ions in the solutions; V is the volume of the heavy-metal ion solution (L); and m is the mass of the tested GGM sample.

The competitive adsorption experiments were conducted using the same amount of the initial or oxidized GGM sample (25 mg), which was added to 8 mL of an aqueous solution containing a mixture of Fe^2+^, Cu^2+^, Cd^2+^, or Pb^2+^ metal ions. This mixture contained 2 mL of each HM stock solution with a concentration of 500 mg/L, resulting in their initial concentration in the competitive experiment of up to 125 mg/L. The other conditions of the tests remained unchanged and the AC was determined to be similar to that in the abovementioned equation.

The specific metal selectivity (%), defined as the ratio of the specific metal concentration that was adsorbed during the competitive experiment to the concentration of all metals adsorbed [24] by the initial and oxidized GGM samples. For example, the specific metal selectivity for one of the investigated heavy-metal ions was calculated as follows:

Selectivity (%) = (C_in_ HM_1_ − C_f_ HM_1_)/((C_in_ HM_1_− C_f_ HM_1_) + (C_in_ HM_2_ − C_f_ HM_2_) + (C_in_ HM_3_ − C_f_ HM_3_) + (C_in_ HM_4_ − C_f_ HM_4_)) (6)

where C_in_ (mg/L) and C_f_ (mg/L) are the initial and final concentrations of the investigated heavy-metal ions.

### 2.12. Statistical Processing of Data

According to the standard practices and analytical methods, all of the measurements of the potentiometric titration, spectrophotometric evaluation of uronic acids, determination of the antioxidant activity, and heavy-metal affinity tests were conducted in triplicates. The collected data were statistically processed by using Statgraphics Centurion XVI, DOE block (Design of Experiment) software (v. 16.2.04, Statpoint Technologies Inc., Warrenton, VG, USA).

## 3. Results

### 3.1. Galactoglucomannan Oxidation Dynamics

**Figure 1:**
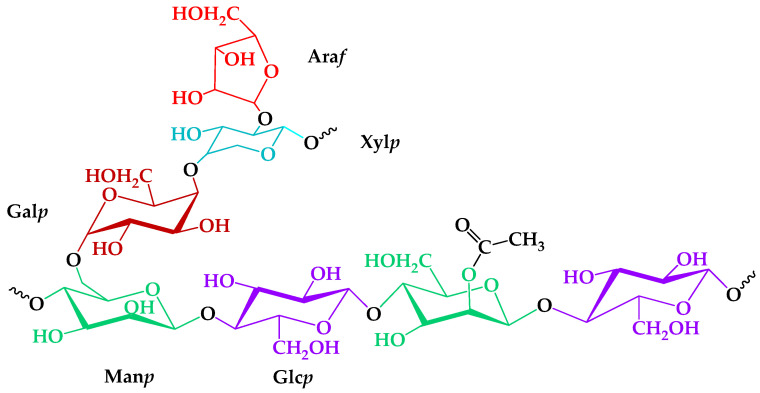
The proposed structure of isolated galactoglucomannan elementary units.

The role of the heterogeneous nature of an isolated GGM was investigated from TEMPO-catalyzed oxidation mechanism perspectives. Analysis of the GGM monosaccharide composition, according to a proven method [27,28], revealed the presence of large amounts of mannose, galactose, and glucose, with some degree of arabinose and xylose in a ratio of 5:3:2:1:1. Trace amounts of the xylose and arabinose could be caused by the presence of an arabinogalactan fraction, meaning that not all of the Gal*p* connected with the Man*p* [32]. An important structural feature was the mannose and glucose residues partially substituted by the acetyl groups—one per three–four hexose units, on average. In summary, according to the registered GC analysis data, a proposed structure of the GGM elementary units is represented in Figure 1.

Considering the moment of the addition of the total volume of the NaClO solution as a starting point for the reaction, a pH-declining rate of oxidation, catalyzed by NaOH consumption, reflects its kinetics. It is generally accepted that the expense of the NaOH could be partially considered an indirect and effective method for comparing the TEMPO-oxidation rates [33]. In addition, the physicochemical properties of GGM-T, as a polysaccharide derivative, depend on the amount of the charged groups caused by carbohydrates with carboxylic acids (uronic acids) [34], which could be evaluated by hydrolysis.

**Figure 2:**
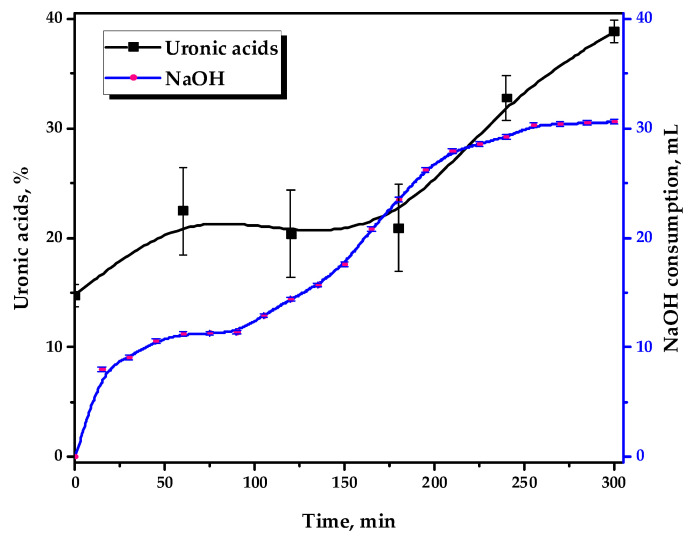
The 0.1 M NaOH consumption by the reaction media and its uronic acid content during the TEMPO-catalyzed oxidation.

Figure 2 depicts the kinetics of 0.1 M NaOH consumption and the uronic acid content, formed by the strong acid hydrolysis of the reaction media in the course of the TEMPO-catalyzed oxidation.

It has been demonstrated that GGM oxidation occurs in stages, with variable dynamics. This could also be indirectly traced by the performed uronic acid content determination in the reaction medium hydrolysates using the modified “carbazole” method [30]. It has been revealed that during the oxidation of GGM that the uronic acid content in its reaction medium hydrolysates increases from 14.8 to 38.9%, which is achieved as a result of a 5 h reaction.

**Figure 3:**
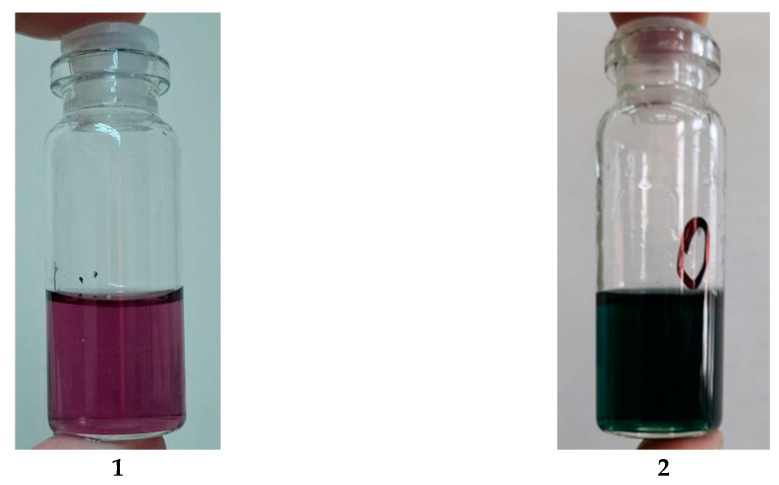
The reaction media hydrolysates, mixed with a carbazole solution: (**1**) “normal” type of the carbazole–uronic acid complex; (**2**) an atypical color change in the carbazole–uronic acid complex.

However, while assessing the uronic acid content in the reaction media of the oxidizing GGM samples using the modified “carbazole” method, atypical changes in the hydrolysates’ color is observed. During the starting periods until 3 h of reaction, the hydrolysates are characterized by a dark-green color instead of violet/purple (Figure 3), typical for carbazole–uronic acid complexes. This fact probably could be a reason of a significant error in the spectrophotometric determination of the uronic acid content in the GGM oxidizing reaction media hydrolysates.

It should be mentioned that this fact is only important for understanding the underlying mechanisms of the TEMPO-catalyzed GGM oxidation. Once the third hour of the reaction is reached, this anomaly is not observed and has no visible effect on the resulting product, so the investigated hydrolysates acquire a stable violet color. Obviously, the nature of this anomaly is most likely related to the specific structural features of the initial GGM: NaOH consumption increases at first and then reaches a plateau, observed in a range of 60–90 min.

Primarily, this could be explained by the oxidative cleavage during the TEMPO-catalyzed oxidation of the arabinoxylose (lacking primary hydroxyl groups) side chains. Its presence adversely affects carboxylate group formation by limiting chemical accessibility [33,35,36]. After 90 min of the oxidation, NaOH consumption by the reaction media continues and gradually reaches a novel plateau after 3–4 h of the reaction, which remains practically unchanged past this point.

**Figure 4:**
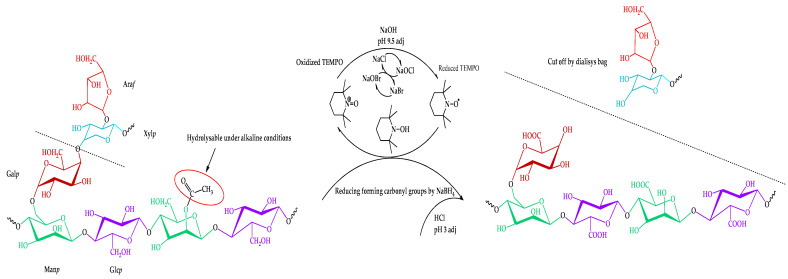
Scheme of the TEMPO-catalyzed oxidation of galactoglucomannan.

Thus, the proposed route of the TEMPO-catalyzed oxidation of the isolated galactoglucomannan is shown in Figure 4.

The resulting mass yields of the product after the separation of the byproducts and inorganic salts by a cut-off dialysis bag vary in a range of 78.3 ± 6.4 wt.%. The obtained yields remain challenging due to the heterogeneous nature of the initially isolated galactoglucomannan.

### 3.2. Determination of Carboxylic Acid Group Content

**Figure 5:**
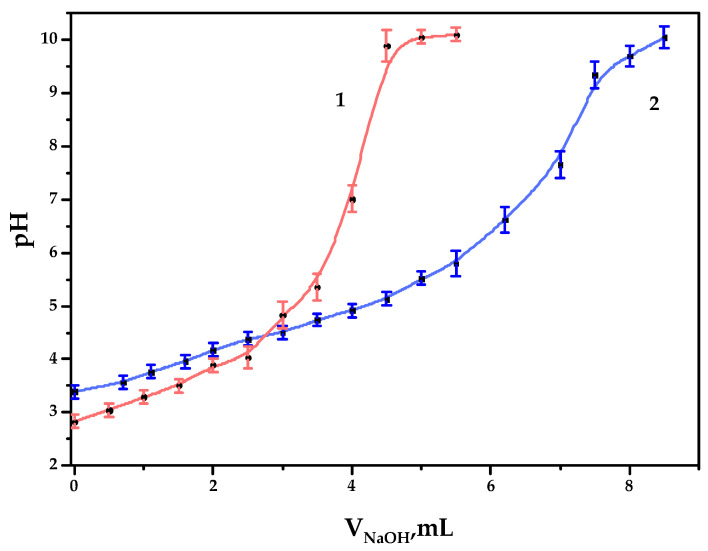
The potentiometric titration curves of the galactoglucomannan samples: (**1**) GGM initial; (**2**) GGM-T.

As a result of the TEMPO-catalyzed GGM oxidation, the formation of carboxylic acid groups on the polysaccharide is expected, which could be estimated by potentiometric titration, expressed by the curve (Figure 5).

As it could be observed, 4.0 and 6.7 mL of NaOH were spent on the titration of GGM and GGM-T samples, respectively. Since the alkali could be spent not only on reaction with carboxyl groups but also on the neutralization of pre-added hydrochloric acid, we therefore subtract 1 mL from V_2_, which results in 3.0 and 5.7 mL of alkali consumption by the sample. Consequently, the concentration of the acid groups in the GGM and GGM-T solutions in terms of the monobasic acid was 0.30 ± 0.01 and 0.57 ± 0.02 mmol/L, respectively. In terms of the carboxylate group content in 1 g of the studied GGM or GGM-T sample polysaccharide, COO- represents up to 0.044 ± 0.001 and 0.084 ± 0.002 g, respectively (4.4 ± 0.1 and 8.4 ± 0.2 wt.%).

### 3.3. FTIR Spectra of Initial and Oxidized GGM Samples 

**Figure 6:**
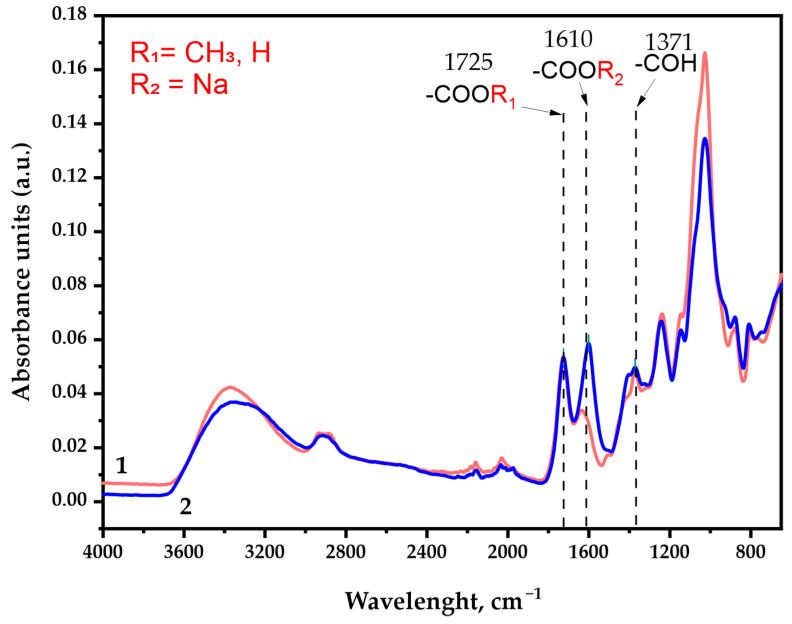
The FTIR spectra of the galactoglucomannan sample absorbance units: (**1**) GGM initial; (**2**) GGM-T.

The FTIR spectra of the GGM samples represented in Figure 6 contain all of the bands characterizing polysaccharides, in which the following specific features should be distinguished. A detailed assignment of the characteristic absorption bands is given in Table S1.

As was expected, the IR spectra depict characteristic broad absorption bands in the region of 3400 cm^−1^, commonly associated with the stretching vibrations of the O-H bonds. The 2900 cm^−1^ region absorption bands could be attributed to the stretching vibrations of the aliphatic -CH_2_ groups [37] connected with the primary alcohols. Its intensity changes insignificantly in the course of the TEMPO-catalyzed GGM oxidation.

The observed intensity perseverance of bands in the ~1725 cm^−1^ region corresponds to vibrations of the alkyl esterified carboxyl groups [38], typical both for the initial and oxidized GGM. In fact, this region could possibly be assigned to the C=O stretching vibration and the C- due to acetyl partial substitution of the mannose and glucose residues in the initially isolated sample. It has already been reported that spruce wood galactoglucomannan is characterized by the high degree of acetylation [39,40]. Nevertheless, the presence of C=O acetyl stretching in the oxidized GGM-T sample was completely excluded by the NaBH_4_ specific reduction, affecting only the carbonyl, not the carboxyl groups.

The most important difference between the initial and oxidized GGM was the significant increase in the band intensity in the ~1610 cm^−1^ region, which was attributed to the asymmetric vibration of the formed -COONa groups as a result of the suggested selective oxidation [37,41] of primary hydroxyl groups of Man*p*/Glc*p*/Gal*p* residues in the initial GGM. Moreover, the a.u. at this region could also be connected with the formation of intra- and intermolecular hydrogen bonds with water [37,42], which also indicates the high presence of carboxylic acid salts in the resulting product.

The ~1370 cm^−1^ region is the “fingerprint” zone for all plant-type polysaccharides. The mutual presence of the a.u. in this region is related to the C–OH deformation vibrations with the O–C–O bonds symmetrical vibrations caused by the high-uronic-acid-carboxylate group’s contribution [42]. The uronic acids are commonly present in the isolated spruce GGM structure in trace amounts, and, as could be observed, visually transform in the case of oxidized GGM.

The absorption band in the 1250 cm^−1^ region could be associated with a variety of modeling compounds containing the C–H bonds. However, the most probable are the C–H planar deformation vibrations of the alkyl functional groups [37,42], obtained as a result of precipitation at the end of the reaction via quenching with an ethanol.

Wide absorption bands in the 1150–1040 cm^−1^ region indicate the C-O-C stretching vibration presence characterizing the glycosidic bonds, inherent for GGM units [43]. As was already mentioned above, these functional groups could undergo rearrangement due to the partial arabinoxylose cleavage in a polar medium during the TEMPO-catalyzed oxidation. Obviously, such decay lowers the intensity of the a.u. in this region for the oxidized GGM. At the same time, the a.u. in the regions of 900 cm^−1^ and 780 cm^−1^ corresponds to the signals of stretching vibrations characterizing the nature of β-galactopyranose and β-glucopyranose bonds [37,44,45]. These regions are common for both the initial and oxidized GGM, witnessing mild oxidation take over with the preservation of the C-O-C bonds [37] crosslinking the “skeleton” of the polysaccharide.

### 3.4. NMR Spectra of Initial and Oxidized GGM Samples

**Figure 7:**
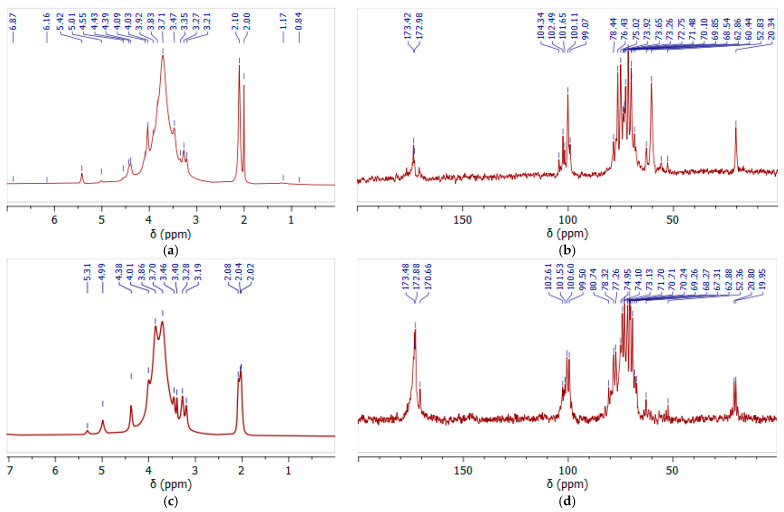
The ^1^H (**a**,**c**) and ^13^C (**b**,**d**) NMR spectra of initial (**a**,**b**) and oxidized (**c**,**d**) GGM recorded in D_2_O.

Still, as a result of GGM isolation from spruce sawdust via oxidative delignification with further ethanol precipitation, side processes transform the functional groups of its terminal units. The subsequent TEMPO-catalyzed oxidation of GGM mainly affects the hexose C-6 groups, converting the primary hydroxyl functional groups into carboxyl groups (Figure 4). Moreover, the quenching of the TEMPO-catalyzed oxidation of the GGM using a mineral acid could also provoke changes in the resulting product. Thus, to evaluate the changes in the structure of the initial and oxidized GGM functional groups, NMR spectroscopy was performed (Figure 7).

In the ^1^H NMR spectrum (Figure 7a) of the initial GGM, the main proton signals characteristic for polysaccharides of this type were determined (H-2, 3.7 ppm; H-3, 3.9 ppm; H-4, 4.1 ppm; and H-5, 4.4 ppm) [46]. The hydrogen atoms, H-1, of the galactosyl and mannosyl residues are separated in the spectrum, and correspond to the signals at 5.3 and 5.0 ppm, respectively [47].

Similar results are observed in the ^13^C spectra, where the main carbon atoms of the polysaccharide were identified (Figure 7b). The significant differences in the proton spectra of the samples are observed in the 20 ppm region (Figure 7a,c). In the initial GGM the two bands are observed, probably corresponding to the methoxyl and ethoxyl protons, indicating a carboxyl group’s esterification. After the TEMPO-catalyzed oxidation, these peaks are preserved, but their intensity lowers, obviously due to the hydrolysis of the ester bonds.

Moreover, this was also confirmed by the signal intensity changes at 60.44 ppm and 62.86 ppm, characterizing the methoxyl and ethoxyl groups’ presence, respectively. It is obvious that after the TEMPO-catalyzed oxidation its intensity significantly decreases, confirming the hypothesis of partial hydrolysis of the bonds (Figure 7b,d). At the same time, intense new signals appear in the 170–174 ppm region, corresponding to the C6 units of the uronic acids (mannuronic, galacturonic, and glucuronic) in esterified and protonated form [48]. The overlapping signals at δ 99–104 ppm in the anomeric region were attributed to the C-1 units of hexoses in the initial GGM, and after the TEMPO-catalyzed oxidation this region changes due to the increase in uronic acid signals.

### 3.5. Molecular Weight Distribution Characteristics of Initial and Oxidized GGM

**Figure 8:**
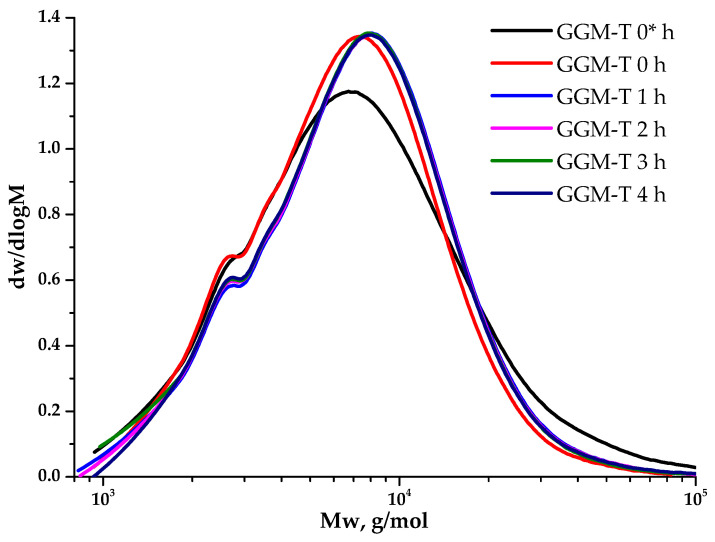
The dynamic molecular weight distribution of the reaction medium of the TEMPO-catalyzed GGM. *—before NaOCl addition.

Polysaccharides’ molecular weight characteristics are crucial, determining many properties of such complexed systems. The observation of the GGM TEMPO-catalyzed oxidation (Figure 8) kinetics from the point of view of molecular weight changes on a polymer basis allows for controlling the reaction course. Moreover, its evaluation aids in selecting the experimental conditions that preserve the initial polymer structure into the future.

As could be observed, the initial GGM is characterized by a monomodal MWD curve profile with Mw of 11,203 g/mol and polydispersity of 2.43. The analysis of the reaction medium, containing TEMPO and NaBr, immediately after adding NaOCl to the mixture (GGM-T 0 h), reveals the transformation of polymer MWD. It is mainly connected with the formation of an intermediate complex compound. At this stage, an increase in the proportion of the low-molecular-weight component is observed, resulting in an overall Mw decrease to 8744 g/mol and polydispersity to 1.59 (Table 1). After 1 h of the TEMPO-catalyzed oxidation, the product MWD profile remains virtually unchanged, but shifts toward the higher-molecular-weight region. Such a change may indicate the polymer structure preservation, inherited from the initial GGM. At the same time, an increase in mass subsequently occurs due to the selective hydroxyl groups’ oxidation. Nevertheless, the oxidation duration from 1 to 4 h does not affect the product MWD, possibly connected with the oxidation already completed in the first hour, which partially corresponds with the data, described in previous sections.

**Figure 9:**
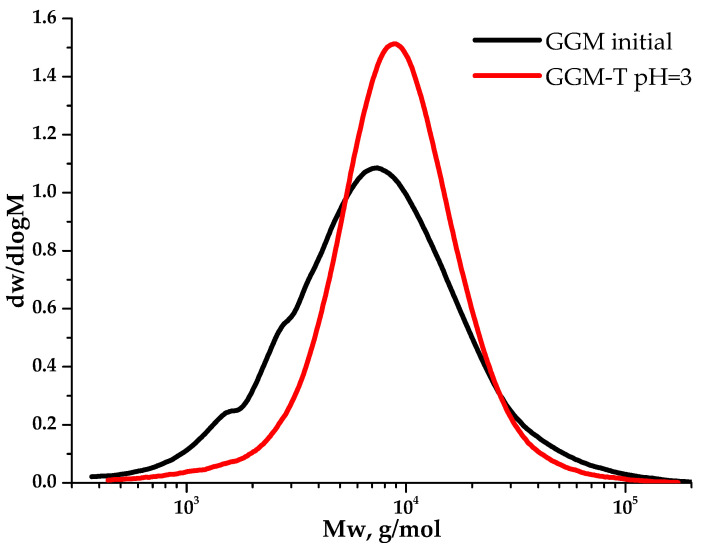
The molecular weight distribution of the galactoglucomannan samples.

Despite a slight decrease in the mass yields of the target substances, the removal of the low-molecular-weight reaction byproducts from the reaction medium is an important stage for oxidized GGM isolation, affecting its quality and performance characteristics, which can be described further. The purge was carried out using a dialysis cut-off bag with a pore diameter of 3.5 kDa (Section 2.2.). Figure 9 depicts the MWD profiles for both the initial and oxidized GGM samples.

### 3.6. Thermal Decomposition Properties of the Initial and Oxidized GGM Samples

**Figure 10:**
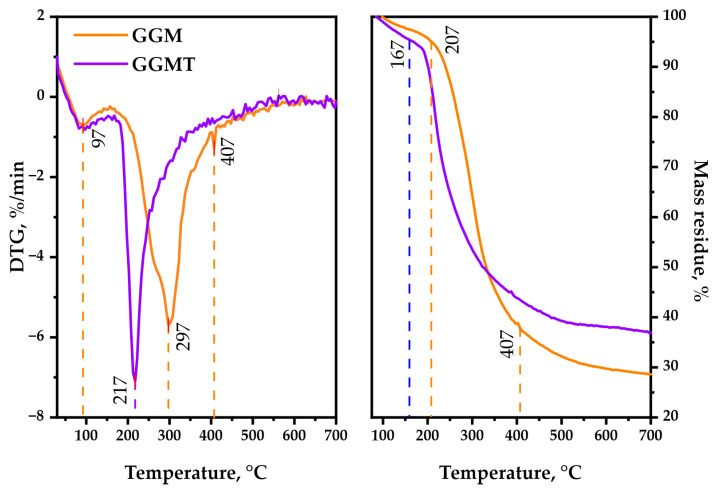
TGA/DTG thermal degradation profiles of the initial and oxidized GGM samples.

As is observable, the thermal decomposition behavior of polysaccharides could reveal its heterogeneous nature by characterizing the underlying chemical reactions that proceed. Simultaneous bond degradation could encourage changes in the polysaccharide conformation and phase conversion, which is crucial to determine the possible area of application for the biopolymer-based materials [49]. The TGA/DTG curves, represented by Figure 10, are registered for the GGM and GGM-T in the 30–700 °C range, where the following common and different features of the observed thermal events should be mentioned.

The first stage (approximately at 92 °C) of thermolysis is connected with the evaporation of sorbed and crystallized moisture by the GGM samples [50], which typically leads to an insignificant change in weight loss. The temperature for 1 wt.% loss of the oxidized sample is 97 °C, whereas for the initial sample it was 117 °C. A further increase in the temperature, approximately up to 207 °C, has almost no effect on the initial GGM structure. It reaches 5 wt.% loss at that point, which could be explained by the hydrogen bond decay of the polar functional groups of polysaccharides. The oxidized GGM-T reaches that value earlier, at 167 °C.

As could be observed, the second stage of initial GGM thermolysis (217–407 °C) reaches 10 wt.% loss at 237 °C, with the maximum at 297 °C followed by intensive mass loss of up to 37.8 wt.% at the end of the stage. Meanwhile, the GGM-T decomposition starts earlier, resulting in 10 wt.% loss at 202 °C, reaching the maximum decomposition rate at 217 °C. Nevertheless, as with its initial form, the GGM-T thermolysis slows down at 407 °C with a 43.5 wt. % mass loss of the residue.

The third stage of the initial GGM decomposition (407–700 °C) is characterized by less weight loss, which corresponds to its carbonization processes, leading to the formation of coke residue [51], resulting in 28.6 wt.% mass residue at the end of the thermolysis. The oxidized GGM-T sample degradation slightly differs—the mass of its residue contains up to 36.9 wt.% at the end of the decomposition.

Based on the obtained data, the main-stage thermal decomposition activation energy of the GGM and GGM-T samples was calculated. The initial GGM thermal decomposition range lies in 217–407 °C, with an activation energy value of 37.4 kJ/mol. At the same time, the GGM-T decomposition activation energy in the main stage (197–400 °C) was characterized by a lower values, 18.1 kJ/mol.

Such GGM-T thermal decomposition behavior may be a consequence of the presence of disordered regions, caused by ester group formation [52]. As a result, the degradation process of the GGM-T initiates earlier at lower temperatures compared to the initial GGM, but the ordered regions of the oxidized polysaccharide resist the decomposition, which leads to higher thermal stability with the perseverance of sample mass.

### 3.7. Antioxidant Activity of Initial and Oxidized Galactoglucomannan

Due to their non-toxic nature, polysaccharides, derived from plant sources, are useful in terms of their antioxidant activity. DPPH and hydroxyl radical scavenging assays are among the most commonly used methods for assessing antioxidant activity. Early studies have shown that the antioxidant properties of plant polysaccharides depend on their structural features, including the composition and ratio of the monosaccharide’s structure, molecular weight characteristics, uronic acid content, and other factors [53].

Antioxidant activity is commonly expressed in terms of IC_50_ values (mg/mL)—the concentration of half-maximal radical inhibition, i.e., the concentration of an antioxidant required to reduce the concentration of radicals by 50% [54]. The IC_50_ values for the GGM and GGM-T samples with respect to DPPH and hydroxyl radical scavenging are represented in Table 2.

**Figure 11:**
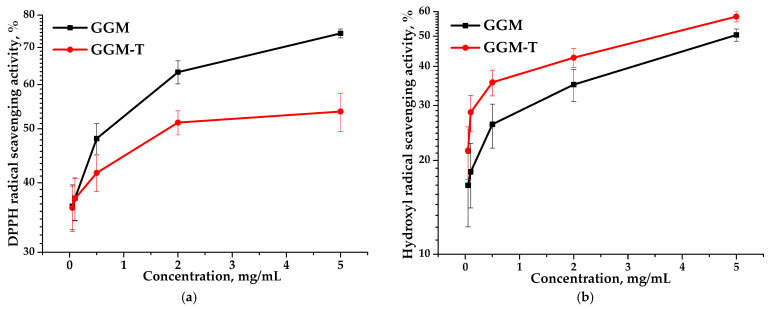
Ability of the GGM and GGM-T samples to scavenge (**a**) DPPH and (**b**) hydroxyl radicals.

Based on the data, it is evident that the IC_50_ values obtained for the GGM and GGM-T samples differ significantly. The IC_50_ of the DPPH for GGM-T is twice as high as that for GGM (Table 2). However, GGM-T is characterized by lower scavenging activity, expressed by a lower IC_50_ value, which is undoubtedly affected by the GGM structure transformation during the TEMPO-catalyzed oxidation. Figure 11 clearly demonstrates that, regardless of the method used for assessing the antioxidant capacity, the scavenging of the DPPH and hydroxyl radicals is dose-dependent. Nevertheless, the presence of the reactive hydroxyl groups in the initial GGM structure, as well as a lower molecular weight (11,203 g/mol), are not the only sources for the high AOA. A more branched structure of the initial GGM, in comparison to the oxidized derivative, is obviously the reason for the greater scavenging capacity for the DPPH free radicals, reaching a maximum of ~74% (Figure 11a). However, such features introduce some limitations into the scavenging of hydroxyl radicals (Figure 11b).

Despite the fact that the oxidized GGM has a higher molecular weight (11,407 g/mol) compared to the initial GGM, the transformation of the polymer structure significantly affected the ability to scavenge free radicals, reaching a maximum of 53% (Figure 11a). It is important to note that the higher carboxyl group content of GGM-T formed by oxidation significantly increases its ability to scavenge hydroxyl radicals from 50 to 58% (Figure 11b).

Thus, it was found that the efficiency of radical scavenging by the oxidized GGM was directly connected with the presence of certain functional groups and inherent structural features. Additionally, the obtained results of AOA assays undoubtedly indicate the prospects for the application of plant polysaccharides and their derivatives in medicine and pharmacology.

### 3.8. Impact of TEMPO-Catalyzed Oxidation on GGM Heavy-Metal Adsorption Capability

Heterogeneous structure polysaccharides, such as GGM, abundant with a number of functional groups on their backbones, could provide numerous binding sites to adsorb the heavy-metal ions in aqueous solutions [23,24]. The most common functional groups that could be involved in adsorption processes are the primary and side hydroxyl groups of the GGM elementary units. It was expected that the presence of insignificant amounts of the acetylated groups in the initial GGM would not impact the adsorption processes. On the other hand, the acetylated groups are subjected to alkaline hydrolysis [55], which could occur in the course of TEMPO-mediated oxidation. It was expected that the following possible acetylated group conversion into hydroxyl groups could enhance the adsorption capacities of its oxidized derivative.

It is important to mention that solution pH is among the most crucial factors affecting a substance’s adsorption properties. A pH = 4 of the tested GGM sample solution was chosen, according to data that report better HM adsorption capacities in the pH = 2–5 range. First of all, it is directly connected with HM ion hydrolysis and its conversion into forms such as Pb_2_(OH)^3+^, Pb_3_(OH)^4+^, and Cd(OH)^+^ [56,57], which negatively affect the absorption capacity.

**Figure 12:**
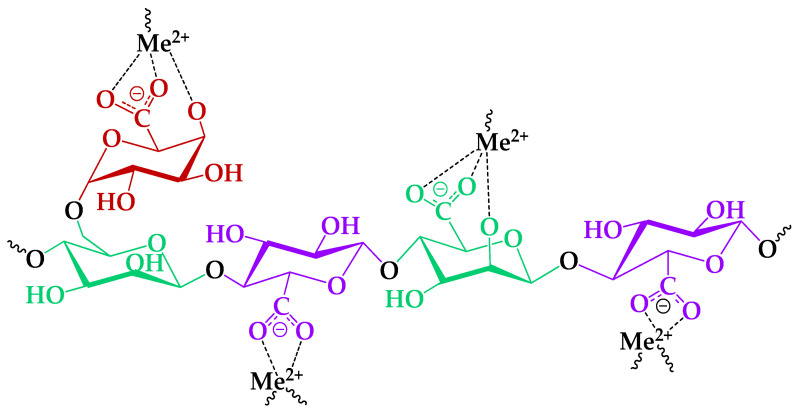
The proposed mechanism of the HM ion adsorption by the GGM-T.

Nevertheless, the cation sorption to the different polysaccharides occurs preferentially via the acidic groups on the latter [24], because of the low steric effect for interaction with metal ions [58]. In this course, consideration of bivalent, specifically HM ion, complexation mechanisms with functional groups of the TEMPO-oxidized GGM was investigated preferably, due to its high affinity (Figure 12).

**Figure 13:**
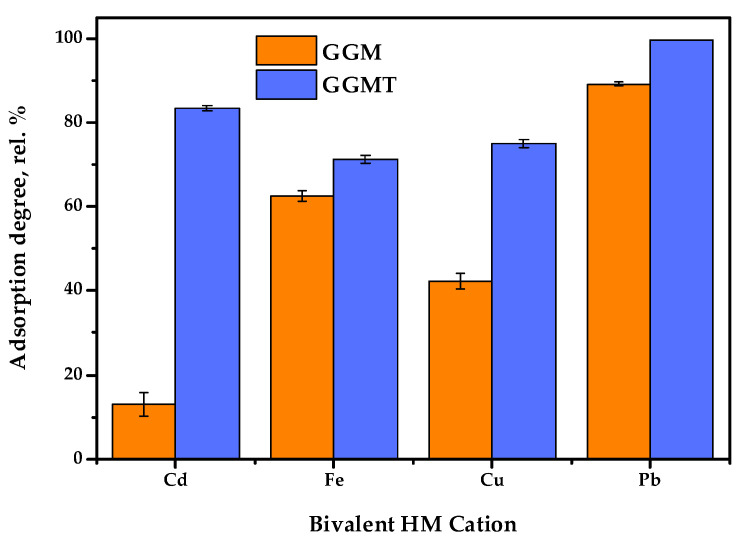
Single heavy-metal ion adsorption degrees of initial and oxidized GGMs.

**Figure 14:**
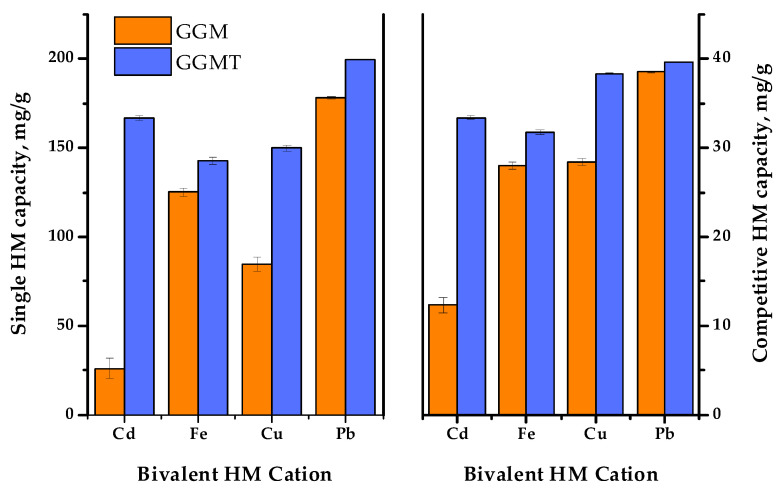
The single and competitive heavy-metal ion adsorption capacities of the initial and oxidized GGMs.

Thus, to investigate the effect of the GGM TEMPO-catalyzed oxidation performed earlier, a series of adsorption experiments were conducted: with single bivalent HM ions and in terms of the competitive tests. The obtained results of the affinity tests are represented in Figure 13 and Figure 14. The numerical values of the sorption properties are represented in Table S2.

The results of adsorption tests in conditions of the single metal ion type used allow us to state that the conducted TEMPO-catalyzed oxidation strongly affects the degree of HM adsorption.

As is shown in Figure 13, the initial GGM was characterized by a low Cd^2+^ affinity with 13.0 rel.% AD, compared to the oxidized derivative, GGM-T, with 83.4 rel.% AD. Expressed in terms of quantity, the AC of Cd^2+^ was valued at 26.0 and 166.8 mg/g for the initial and oxidized samples, respectively. The smaller but significant difference between the initial and oxidized GGM sample AD rates is also observed for Cu^2+^ ions—42.3 and 75.0 rel.%, respectively. The smallest difference in the AD value rates of the initial and oxidized GGM samples is observed for Fe^2+^ and Pb^2+^; nevertheless, the Pb^2+^ adsorption values are approaching the possible maximum, while the specifics of Fe^2+^ adsorption remain unclear, which will be described below.

In order to reveal the adsorption performance of the initial and oxidized GGM samples in a multi-component system, a mixture of the selected HM ions was tested in terms of the competitive experiment. The obtained results are depicted in Figure 14.

As was expected, the summary adsorption capacities for both the initial and oxidized GGM samples in most cases were lower to some extent, compared to the single HM ion AC. Nevertheless, since the same amount of the total HM ion concentrations was used in both single and competitive tests, it is important to note that the summary capacity of the initial GGM in conditions of a competitive experiment was higher than the capacity of single Cd^2+^ and Cu^2+^—107.3 ± 1.7 mg/g compared to 26.0 ± 5.7 and 84.6 ± 3.8 mg/g, respectively. As for the oxidized GGM derivative, its summary capacity in terms of the competitive tests was higher, in comparison to the initial GGM—143.1 ± 0.6 mg/g. However, due to the overall GGM-T greater single HM adsorption capacity, its summary AC values in a competitive experiment exceeds the AC values registered for Fe^2+^. This fact obviously demonstrates a competitive relationship between the investigated bivalent HM ion adsorption.

Noticeably, the relative difference ratio of Fe^2+^ adsorption by the GGM samples in conditions of single HM and competitive experiments has an equaling value. This could be due to the specifics of Fe^2+^ ions, which could interact with water in solutions, or caused by the substitution of hydrogen from the carboxyl groups formed by the TEMPO-catalyzed GGM oxidation. Moreover, it was expected that the Fe^2+^ ion adsorption selectivity would increase at the cost of less active ions such as Cu^2+^, which should be easily substituted, but on the contrary, that has no effect.

As is shown in Figure 14, Pb^2+^ adsorption by both the initial and oxidized GGM samples was predominant in terms of the single HM ion and competitive experiments. It is obvious that GGM was initially inclined to adsorb Pb^2+^ with a high degree of selectivity and good adsorption capacities, even in a mixture with a relatively strong affinity for Pb^2+^, which could be connected with its lower hydration radius [59].

Thus, as has been shown by the obtained data, the oxidized GGM-T adsorbent exhibited superior adsorption properties and affinity for multiple metal ions in terms of the competitive experiments compared to its original, the initial GGM. The relative selectivity of HM adsorption in terms of the competitive tests undergo rearrangement, which was obviously caused by the modification of the initial GGM structure. As can be seen from Table S2, the HM ion adsorption selectivity values of the initial GGM varies in the order of Cd^2+^ < Fe^2+^ ≈ Cu^2+^ < Pb^2+^. Meanwhile, the TEMPO-oxidized derivative GGM-T selectivity values increase in the order of Fe^2+^ < Cd^2+^ < Cu^2+^ < Pb^2+^.

According to the existing data, the cellulose, starch, chitosan, sodium alginate, and their derivatives are traditionally observed as promising adsorbents. As for the polysaccharides, they are most commonly represented by hydrogels, synthesized on a guar gum, agar, or hyaluronic acid basis. It was found that the oxidized GGM-T derivative adsorption capacity of single Cu^2+^ (150.0 mg/g) exceeds alginate/carboxymethyl cellulose (105.93 mg/g) [60] and alginate/polyvinyl/ZIF-9 (98.98 mg/g) [61] materials’ adsorption capacities. As for Pb^2+^, the GGM-T adsorption capacity (199.2 mg/g) also exceeds alginate/carboxymethyl cellulose [60], polyvinyl alcohol/alginate [62], and *E. prolifera*-based double network hydrogel (166.7 mg/g) [63] materials.

Nevertheless, in comparison to synthetic polymer adsorption capacity, GGM-T was characterized by a lower efficiency. One possible path for improving existing adsorption capabilities lies in the application of GGM-T to the synthesis of composite materials. For example, materials based on biodegradable compounds like chitosan/sodium alginate/calcium ion double network hydrogels exhibit greater Pb^2+^, Cu^2+^, and Cd^2+^ affinities in terms of the competitive tests—176.50, 70.83, and 81.25 mg/g, respectively [64].

The applications of such biopolymers could vary significantly, based on the obtained results. It is obvious that oxidized GGM-T has great potential in the synthesis of composite materials with multiple functional groups or polyelectrolyte complexes, also used as drug carriers [65]. Such affinity to the cations, combined with high AOA and excellent water solubility, allows for proposing oxidized GGM in trials of HM uptake from living organisms. Due to the low toxicity of the polymer basis, metal complexes represent potential for the development of products used as preparation for the protection of skin and hair from the harmful action of UV radiation [66]. In addition, the complexation of polysaccharides with cations is applicable in analytical methods of HM determination, with its easy removal achievable via centrifugation and filtration. Moreover, such properties could find their application in the field of electrodialysis and reverse osmosis processes for the removal of heavy metals from aqueous solutions.

## 4. Conclusions

A novel bio-based polymer was synthesized from isolated spruce galactoglucomannan via TEMPO-catalyzed oxidation. The dynamics of the process were controlled using the well-known titration, gel permeation chromatography, and spectrophotometric methods. It was observed that GGM oxidation proceeds in a mild way; nevertheless, the partial cleavage of glycoside bonds linking arabinoxylose’s units, the presence of which is due to the techniques used for GGM isolation, was also confirmed by FTIR. Despite a slight decrease in mass yields of the oxidized GGM (78.3 ± 6.4 wt.%), the removal of the low-molecular-weight reaction byproducts is an important stage for target substance isolation from the reaction medium, affecting both quality and performance characteristics.

It has been revealed that during the oxidation of GGM, the uronic acid content multiplies by more than twice (from 14.8 to 38.9%), which indirectly points to oxidation dynamics, corresponding with the NaOH consumption rate during the process. Expressed by a quantity, the carboxylate group content in the polysaccharide contains up to 8.4 wt.%, indicating the efficiency of the conducted TEMPO-catalyzed oxidation.

As was expected, the thermal degradation of the oxidized GGM-T begins earlier at lower temperatures compared to the initially isolated sample. Nevertheless, we assume that the ordered regions of the oxidized polysaccharide resist decomposition, which leads to a higher thermal stability, in general, with a greater mass perseverance.

In this study, the initial and oxidized GGMs were successfully tested to adsorb various bivalent HMs, singled or in terms of the competitive tests (Fe^2+^, Cu^2+^, Cd^2+^, or Pb^2+^). The adsorption capabilities of the initial and oxidized GGM were determined by the physical crosslinking of polysaccharide chains with HM ions. The obtained complexes were further precipitated by ethanol and easily separated from the solution as a hydrogel via centrifugation and filtration through a PTFE membrane. It was found that the HM ions’ adsorption capability of the initial GGM was mainly influenced by the hydroxyl-HM ions’ migrating activity and varies in the order of Cd^2+^ < Fe^2+^ ≈ Cu^2+^ < Pb^2+^. The presence of the acetylated groups in the isolated initial GGM insignificantly affects adsorption properties. Additionally, the TEMPO-oxidized derivative GGM-T adsorption capability is mostly influenced by the presence of both the hydroxyl and formed carboxyl groups. Their presence modifies the HM ions’ migrating activity, which increases in the order of Fe^2+^ < Cd^2+^ < Cu^2+^ < Pb^2+^. In accordance with the existing data, the GGM-T derivative adsorption capacity exceeds a number of traditionally studied HM adsorbent materials. Nevertheless, the obtained bio-based polymer is still inferior in comparison to the synthetically obtained adsorbents in terms of the competitive adsorption tests, which constitutes a new challenge. The synthesis of composite materials based on GGM-T is a possible route with which to improve the biopolymer properties, which should be considered for the further studies.

## Figures and Tables

**Table 1:** Molecular weight characteristics of the GGM samples.

Scheme	M_p_ (g/mol)	M_n_ (g/mol)	M_w_ (g/mol)	PD
GGM initial	7376	4611	11,203	2.43
GGM-T pH = 3	8945	6716	11,407	1.70
GGM-T 0 * h	6692	5711	10,595	1.86
GGM-T 0 h	7451	5510	8744	1.59
GGM-T 1 h	8047	5913	9639	1.63
GGM-T 2 h	8047	5863	9588	1.63
GGM-T 3 h	7864	5830	9457	1.62
GGM-T 4 h	8047	5855	9694	1.66

*—before NaOCl addition.

**Table 2:** The GGM and GGM-T samples’ IC_50_ values in relation to DPPH and hydroxyl radical scavenging.

Sample	IC_50_ Values (mg/mL)	
	DPPH	•OH^−^
GGM	1.28	4.71
GGM-T	3.26	3.54

## Data Availability

All data generated during this study are included in the article.

## Supplementary Materials

The following supporting information can be downloaded at: https://www.mdpi.com/article/10.3390/antiox14050569/s1, Table S1: Absorbance units of initial and oxidized GGM registered by FTIR; Table S2: Sorption properties of initial and oxidized GGM with various bivalent heavy metals.
