# Supplementary material for: TEMPO-Oxidized Spruce Galactoglucomannan–Biopolymer with Enhanced Antioxidant Activity and Selective Heavy-Metal Sorption
Source: Antioxidants (Basel). 2025 May 9;14(5):569. doi: 10.3390/antiox14050569 (PMC12108164; doi:10.3390/antiox14050569)
Supplement: Supplementary file 1 [file antioxidants-14-00569-s001.zip › antioxidants-3574997-supplementary.pdf]

antioxidants

MDPI

Article

TEMPO-Oxidized Spruce Galactoglucomannan–Biopolymer with Enhanced Antioxidant Activity and Selective Heavy Metals Sorption

Vladislav A. Ionin[1,2], Yuriy N. Malyar[1,2,*], Valentina S. Borovkova[1,2], Dmitriy V. Zimonin[1,2], Alexandr S. Kazachenko[1,2,3]

1 Institute of Chemistry and Chemical Technology, Krasnoyarsk Science Center, Siberian Branch Russian Academy of Sciences, Akademgorodok 50/24, Krasnoyarsk, 660036 Russia; ionin.va@icct.krasn.ru (V.A.I.); yumalyar@gmail.com (Y.N.M.); bing0015@mail.ru (V.S.B.); zimonind89@mail.ru (D.V.Z.); askazachenko@sfu-kras.ru (A.S.K.)

2 School of Non-Ferrous Metals, Siberian Federal University, pr. Svobodny 79, Krasnoyarsk, 660041 Russia.

3 Reshetnev Siberian State Univ Sci & Technol, Inst Chem Technol, Mira St 82, Krasnoyarsk 660049, Russia

* Correspondence: yumalyar@gmail.com; Tel.: +7-9082065517.

Supplementary Materials:

Table S1. Absorbance units of initial and oxidized GGM registered by FTIR-spectroscopy

Wavelength, cm^{-1}	Comment
3600-3200	Stretching vibrations of O-H
2900-2800	Stretching vibrations of aliphatic $-CH_2$
1750-1725	Stretching vibrations of aliphatic C=O or C-O
1610-1600	Asymmetric stretching vibrations of -COONa or intra- and intermolecular hydrogen bonds
1400-1300	Stretching vibrations of C-OH
1250	Stretching vibrations of C-H
1150-1040	Stretching vibrations of O-C-O

Table S2. Sorption properties of initial and oxidized GGM with various bivalent heavy metals

HM	Adsorption degree of single HM ions, rel. %		Single HM ions capacity, mg/g		Competitive HM ions capacity, mg/g		Selectivity, rel. %	
	GGM	GGM-T	GGM	GGM-T	GGM	GGM-T	GGM	GGM-T
Cd^{2+}	13.0±2.9	83.4±0.6	26.0±5.7	166.8±1.1	12.3±0.9	33.4±0.2	11.5±0.7	23.3±0.1
Fe^{2+}	62.6±1.2	71.4±0.9	125.2±2.5	142.8±1.9	28.0±0.4	31.8±0.3	26.1±0.1	22.2±0.1
Cu^{2+}	42.3±1.9	75.0±0.8	84.6±3.8	150.0±1.7	28.4±0.4	38.4±0.1	26.5±0.1	26.8±0.1
Pb^{2+}	89.2±0.4	99.6±0.1	178.4±0.7	199.2±0.1	38.5±0.1	39.6±0.1	35.9±0.6	27.7±0.1
		Σ			107.3±1.7	143.14±0.6		
